# Identifying First-Trimester Risk Factors for SGA-LGA Using Weighted Inheritance Voting Ensemble Learning

**DOI:** 10.3390/bioengineering11070657

**Published:** 2024-06-27

**Authors:** Sau Nguyen Van, Jinhui Cui, Yanling Wang, Hui Jiang, Feng Sha, Ye Li

**Affiliations:** 1 Shenzhen Institute of Advanced Technology, Chinese Academy of Sciences, Shenzhen 518055, China; saunv@siat.ac.cn (S.N.V.); h.jiang@siat.ac.cn (H.J.); 2 University of Chinese Academy of Sciences, Beijing 100040, China; 3 Faculty of Basic Sciences and Foreign Languages, University of Fire Fighting and Prevention, Hanoi 100000, Vietnam; 4 Department of Obstetrics and Gynecology, The Third Affiliated Hospital of Sun Yat-sen University, No. 600, Tianhe Road, Guangzhou 510630, China; cuijhui@mail.sysu.edu.cn; 5 Department of Anesthesiology, The Third Affiliated Hospital of Sun Yat-sen University, No. 600, Tianhe Road, Guangzhou 510630, China; wangyl5@mail.sysu.edu.cn; 6 Hangzhou Institute of Advanced Technology, Hangzhou 310000, China

**Keywords:** SGA (small for gestational age), LGA (large for gestational age), AGA (appropriate for gestational age), machine learning, first trimester ending

## Abstract

The classification of fetuses as Small for Gestational Age (SGA) and Large for Gestational Age (LGA) is a critical aspect of neonatal health assessment. SGA and LGA, terms used to describe fetal weights that fall below or above the expected weights for Appropriate for Gestational Age (AGA) fetuses, indicate intrauterine growth restriction and excessive fetal growth, respectively. Early prediction and assessment of latent risk factors associated with these classifications can facilitate timely medical interventions, thereby optimizing the health outcomes for both the infant and the mother. This study aims to leverage first-trimester data to achieve these objectives. This study analyzed data from 7943 pregnant women, including 424 SGA, 928 LGA, and 6591 AGA cases, collected from 2015 to 2021 at the Third Affiliated Hospital of Sun Yat-sen University in Guangzhou, China. We propose a novel algorithm, named the Weighted Inheritance Voting Ensemble Learning Algorithm (WIVELA), to predict the classification of fetuses into SGA, LGA, and AGA categories based on biochemical parameters, maternal factors, and morbidity during pregnancy. Additionally, we proposed algorithms for relevance determination based on the classifier to ascertain the importance of features associated with SGA and LGA. The proposed classification solution demonstrated a notable average accuracy rate of 92.12% on 10-fold cross-validation over 100 loops, outperforming five state-of-the-art machine learning algorithms. Furthermore, we identified significant latent maternal risk factors directly associated with SGA and LGA conditions, such as weight change during the first trimester, prepregnancy weight, height, age, and obstetric factors like fetal growth restriction and birthing LGA baby. This study also underscored the importance of biomarker features at the end of the first trimester, including HDL, TG, OGTT-1h, OGTT-0h, OGTT-2h, TC, FPG, and LDL, which reflect the status of SGA or LGA fetuses. This study presents innovative solutions for classifying and identifying relevant attributes, offering valuable tools for medical teams in the clinical monitoring of fetuses predisposed to SGA and LGA conditions during the initial stage of pregnancy. These proposed solutions facilitate early intervention in nutritional care and prenatal healthcare, thereby contributing to enhanced strategies for managing the health and well-being of both the fetus and the expectant mother.

## 1. Introduction

Small for Gestational Age (SGA) infants, defined as having birth weights below the 10th percentile for their gestational age, constitute approximately 7–10% of newborns globally. This percentage can escalate to 20–30% in developing countries [1,2]. The etiology of SGA is multifactorial, involving maternal factors such as inadequate nutrition, smoking, hypertension, and chronic kidney disease [3]. SGA is associated with an increased risk of adverse perinatal outcomes, including prematurity, neonatal morbidity, developmental delays, and long-term health issues. Severe cases of SGA may result in fetal death during the antenatal period or lead to postnatal complications such as meconium aspiration syndrome, respiratory distress syndrome, hypoglycemia, and metabolic syndrome in adulthood [4].

Conversely, Large for Gestational Age (LGA) infants, with birth weights exceeding the 90th percentile, occur in approximately 5–10% of newborns globally. In certain regions, this prevalence can reach up to 15–30% [1,5,6]. Maternal factors such as gestational diabetes, maternal obesity, excessive weight gain during pregnancy, and genetic predispositions contribute to the development of LGA [7,8]. LGA births pose challenges during labor and delivery, increasing the likelihood of cesarean sections, birth injuries, and metabolic complications for the newborn. Moreover, LGA infants are at an increased risk of obesity, insulin resistance, and hypertension in later life [7,9]. Given its potential impact on maternal and neonatal health, the condition warrants attention in perinatal and pediatric medicine.

SGA and LGA infants present distinct implications for maternal, fetal, and postnatal health, necessitating a comprehensive understanding of the associated risks. SGA infants may progress into severe conditions potentially resulting in fetal demise during the antenatal period or leading to complications such as meconium aspiration syndrome, asphyxia, respiratory distress syndrome, hypoglycemia, hypothermia, bronchopulmonary dysplasia, and hyperviscosity thrombosis during the intranatal period [4]. Postnatally, SGA infants are predisposed to developing metabolic syndrome, obesity, hypertension, diabetes, and coronary heart disease, imposing significant consequences on the affected individuals and their caregivers [10].

LGA infants face heightened risks of short-term complications, including birth trauma, shoulder dystocia during delivery, and an increased likelihood of admission to the neonatal intensive care unit. They may also encounter long-term metabolic and cardiovascular issues such as obesity, insulin resistance, and hypertension [9]. Early detection and intervention are crucial, involving antenatal care, dietary interventions, and glycemic control [8]. A comprehensive list of SGA and LGA implications for mothers, fetuses, and children postbirth is provided in Table 1.

Although many studies have found high risks of SGA and LGA for fetal, infant, mother, and SGA/LGA outcomes during childhood, current studies have some limitations [12,13,14,15,16,17,18,19,20,21,22,23] (detailed in Section 2):

1. Studies mainly concentrate on enhancing the estimation of fetal weight, fetal birth weight, and fetal gestational age from ultrasound images [12,13,15,16,17,18,19,20].
2. Fetuses are categorized based on one or more criteria, including fetal weight, head circumference, abdominal circumference, fetal heart rate, and their integration with other fetal attributes. Alternatively, the classification may be based on fetal weight along with maternal attributes [21,22].
3. Studies often focus on binary classification between SGA/LGA and their respective control groups [21,22].
4. There is a lack of studies exploring hidden risk factors associated with SGA and LGA fetuses beyond isolated risk factors [11,15,23].

Consequently, these limitations raise two critical questions: (1) Is it feasible to diagnose SGA and LGA fetuses before 24 weeks when definitions of SGA, LGA, and AGA are based on fetal weight from 24 weeks to 36 weeks of gestation? (2) What latent risk factors lead to SGA or LGA fetuses?

Therefore, the primary objective of this study is to introduce a machine learning approach aimed at predicting fetal classification into three distinct groups, SGA, LGA, or AGA, based solely on maternal demographics, maternal history factors, and maternal biomarker attributes gathered at the end of the first trimester (around 13 weeks of gestation). Subsequently, this study seeks to assess the significance of these attributes in influencing the developmental trajectory of the fetuses and leading to SGA or LGA outcomes. The key contributions of this research are delineated as follows:

- The first research work on early diagnosis of SGA, LGA, and AGA conditions at the end of the first trimester and only used maternal demographics, maternal history factors, and maternal biomarker attributes.
- Introduction of a novel algorithm designed to validate previous diagnoses of SGA-LGA conditions. This algorithm achieves a classification accuracy (Acc) of 0.92, which surpasses the performance of five state-of-the-art machine learning algorithms. It also demonstrates a lower Mean Squared Error (MSE) in comparison. Furthermore, our testing results include average accuracy and balanced certainty measurement scores of approximately 0.92.
- Proposal of algorithms aimed at identifying latent risk factors potentially associated with the likelihood of an SGA or LGA fetus based on our proposed classifier.

This initiative promises to aid medical practitioners in evaluating clinically relevant patient data, bolstering diagnostic and therapeutic decision-making processes. The proposed solutions have the potential to significantly impact the management of fetal health, particularly in the early stages of pregnancy, thereby contributing to improved health outcomes for both the mother and the fetus.

## 2. Computation Techniques Applied for SGA-LGA Classification and Analysis

In recent years, the application of machine learning (ML) and deep learning (DL) techniques in perinatal medicine has significantly increased. These advanced techniques have proven valuable in diagnosing various fetal and maternal well-being complications, predicting SGA and LGA infants, estimating fetal weight and gestational age, and identifying latent risk factors associated with fetal and maternal health. The multifaceted functionalities of these applications extend beyond mere diagnosis, encompassing a broader spectrum of tasks, including management, treatment, and an enriched understanding of the pathophysiological aspects of perinatal conditions.

In 2015, Harper et al. proposed using ultrasound surveillance for LGA classification [12]. Subsequently, Shen et al. (2017) utilized sonography to estimate fetal weight [13], and Harper et al. integrated abdominal circumference (AC) into the approach [14]. In 2019, Feng et al. employed a Support Vector Machine (SVM) and Deep Belief Network (DBN) to enhance the accuracy of fetal weight estimation and facilitate the identification of potential delivery-related risks by clinicians [15]. In 2021, Jing Tao et al. conducted a comparative analysis employing Convolutional Neural Networks (CNNs), Random Forest (RF), Logistic Regression (LR), Support Vector Regression (SVR), a Back Propagation Neural Network (BPNN), and a proposed algorithm named hybrid-LSTM for predicting fetal birth weight [16].

In 2021, Mobadersany et al. used GestAltNet to estimate gestational age [17]. Moreover, in 2022, Wasif Khan et al. employed Random Forest (RF) for SGA classification and Logistic Regression (LR) with Synthetic Minority Over-sampling Technique (SMOTE) for birth weight estimation [18]. Lee et al. use ultrasound images to apply a ResNet-50-based model to estimate fetal gestational age [19]. YiFei et al. utilized Deep Neural Networks (DNNs) for fetal weight estimation [20].

In 2018, Kuhle et al. compared LR with other ML algorithms such as Elastic Net (EN), Classification Tree (CT), RF, Gradient Boosting (GB), and Neural Networks (NNs) in SGA-LGA classification, maternal characteristics prepregnancy and gestational parameters at 26 weeks were used to predict SGA and LGA, the AUC was poor, ranging from 0.563 to 0.748 [21]. Then, in 2019, Faheem Akhtar et al. introduced a GridSearch-based FRECV+IG feature selection technique and evaluated various ML algorithms, including LR, SVM with an FRB kernel, and Decision Tree (DT) to classify infants as either LGA or Non-LGA with an accuracy of 92% when using historical demographics, maternal attributes, and fetus weights at 24–33 gestational weeks [22].

In 2021, Sau et al. proposed a novel stacking ensemble algorithm to classify SGA and control group with an accuracy of 94.73% and computed feature importance to identify latent risk factors associated with SGA infants in their childhood [11]. Additionally, in 2023, Faiza et al. conducted a study involving multiple ML algorithms, including Naïve Bayes (NB), RF, Bagging, LR, and J48 to identify significant risk factors associated with newborn sepsis with about 88.4% accuracy [23].

In summary, many studies have utilized machine learning (ML) and deep learning (DL) to improve the estimation of fetal weight, birth weight, and gestational age, particularly by using attributes from the late stage of gestation (24 to 36 weeks) or by combining them with maternal attributes to classify fetuses or infants into binary classification SGA or LGA and their respective control categories. However, there is a lack of studies employing ML algorithms to uncover hidden risk factors associated with SGA and LGA. Therefore, there is a gap in predicting multiple classifications into SGA, AGA, and LGA categories before 24 weeks of gestation and identifying latent risk factors leading to SGA and LGA outcomes.

Recently, RF, SVM, CART, XGB, and KNN algorithms have been highly recommended for classifying SGA-LGA data. These algorithms are inherently capable of calculating feature importance for biomedical data. Additionally, significant efforts are being made to develop explainable deep learning (DL) models that outperform traditional classification methods in some complex tasks [24,25,26,27]. However, Rudin Cynthia has argued that we should avoid using DL models for high-stakes decisions [28].

The application of machine learning (ML) and DL in perinatal medicine reflects the significant potential of these advanced methods to enhance the understanding, diagnosis, and management of perinatal conditions. The multifaceted functionalities of these applications promise to revolutionize the field further, contributing to improved health outcomes for both mothers and fetuses.

## 3. Materials and Methods

**Figure 1:**
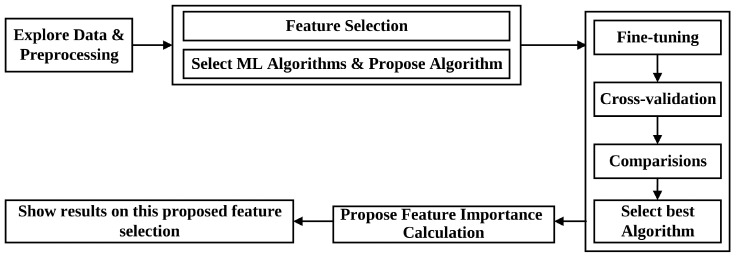
Our methodology for classifying SGA-LGA data.

Figure 1 illustrates our methodology for classifying the SGA-LGA dataset. These steps are important and practical in machine learning. These procedures can be combined and followed according to the steps outlined below:

- Step 1. Data Exploration and Preprocessing: The initial step involves comprehensive data exploration and preprocessing, a fundamental and imperative phase in machine learning. This step aims to clean the dataset, ensuring optimal data utilization while conducting essential preprocessing tasks.
- Step 2. Feature Selection: Feature selection is performed using our proposed feature selection algorithm, domain expertise, and the incorporation of state-of-the-art algorithms. The objective is to identify relevant features, representing our data accurately and effectively enhancing the selected algorithms’ performance.
- Step 3. Algorithm Fine-Tuning and Comparative Analysis: The fine-tuning process is to find the best parameters for each algorithm and then compare algorithmic performances, assessing accuracies and Mean Squared Errors (MSEs) to determine the optimal algorithm for the classification task.
- Step 4. Feature Importance Calculation: Feature importance is calculated based on identifying the most effective or proposed algorithm if it is more outstanding than others. This step involves ascertaining the significance of each feature in contributing to the classification outcomes.
- Step 5. Results Presentation on Feature Importance: The final step entails presenting the results derived from the calculated feature importance. This includes a comprehensive exposition of the contribution of individual features to the classification process.

Further details about these steps are provided in the subsequent sections.

### 3.1. Data and Preprocessing

#### 3.1.1. Dataset

The present research utilizes a dataset comprising 7943 subjects, including 1752 overweight pregnancies (BMI≥24) and 6191 normal or underweight pregnancies (BMI<24). The data were collected from 2015 to 2021 at the Third Affiliated Hospital of Sun Yat-sen University, Guangzhou, China. Inclusion criteria were as follows: (1) aged 17 to 47 years, and (2) intending to deliver at our affiliated hospital. The exclusion criteria were (1) a history of cardiac or hepatic disease and (2) participation in other clinical research. The use of the dataset was approved by ethics approval number “201902-338-01”. The dataset includes 424 SGA (≈5.33%), 928 LGA (≈11.68%), and 6591 AGA (≈82.99%) infants.

This study examines various measurements, encompassing biochemical parameters, maternal demographics, maternal factors, and morbidity during pregnancy. The biochemical parameters include plasma protein A, the free beta subunit of human chorionic gonadotropin (free β-HCG), fasting plasma glucose (FPG), total cholesterol (TC), triglycerides (TG), and high- and low-density lipoproteins (HDL, LDL), measured at 11 to 13 weeks of gestation. Maternal factors incorporate maternal age, obesity, parous, history of Gestational Diabetes Mellitus (GDM), and family history of diabetes.

This study also undertakes several key tasks, such as analyzing the correlation among features related to SGA and LGA from clinical experts. It then selects characteristic groups to reduce data dimensionality for data analysis and grouping. Furthermore, this study applies several state-of-the-art machine learning algorithms and our proposed algorithm to predict SGA, LGA, and normal fetuses. The groups of attributes and a detailed list are presented in Table 2.

#### 3.1.2. Data Preprocessing

Preprocessing is essential before utilizing the dataset to explore correlations and trends, serving as an initial and crucial step in machine learning. This step ensures that we have well-processed data. It involves removing duplicate and invalid data (such as invalid entered value(s) or outliers that fall outside the normal/valid range), addressing missing values (such as through removal or imputation, etc.), and handling imbalanced classification data. Additionally, if the data are high-dimensional, we can employ techniques to reduce its dimensionality, such as automatic feature selection approaches, Principal Component Analysis (PCA), the Pareto rule proposed by Roccetti Marco et al. [29], or our proposed algorithm, as detailed in Section 3.1.3. Special preprocessing methods, such as Zelaya’s proposed method for data sensitive to outliers [30], can also be employed.

After removing 45 duplication/invalid (invalid entered values or values outside the normal range) rows from 7943 rows × 34 columns in the dataset, it is composed of 7898 unique rows consisting of 422 SGA (5.34%), 922 LGA (11.67%), and 6554 AGA (82.99%), respectively. The data contain 1133 rows (≈14.34%) and 11 columns with missing values (for more details, see Table 3).

The objective is to uncover hidden attributes associated with SGA and LGA fetuses by the end of the first trimester. Consequently, instead of employing all features, certain unnecessary features are eliminated, such as *height (cm)* (retaining *height (m)*), or merging diagnosed/labeled *SGA* and *LGA* columns into a single *InfantType* column.

As in Table 3, the percentage of missing values is relatively small (4448/(7943×34)≈1.65%), with 1133/7943 (14.26%) samples containing missing values. However, the number of samples with missing values is not insignificant, predominantly in the *TC, TG, HDL*, and *LDL* columns, while the remaining columns with missing values account for 152 missing values (≈0.056%), which is negligible. Therefore, finding an appropriate approach for processing data is crucial, with steps such as cleaning and maximizing data usage being important for the proposed solution.

There are three primary approaches to handling missing values: imputation (i.e., imputed by mean, median, interpolation, and most frequent), data removal, or employing an algorithm that accommodates missing data. Some algorithms address missing values by learning the optimal imputation values based on the reduction in training loss (i.e., Random Forest), while others will disregard them (i.e., LightGBM). However, certain algorithms will encounter an error when faced with missing data (i.e., Logistic Regression). In this case, the missing data will be handled and cleaned before feeding them to the algorithm. Thus, we employ median imputation in this research to maximize data usage.

Subsequently, the dataset is standardized. In addition to the strategies mentioned earlier, the Synthetic Minority Oversampling Technique (SMOTE) is employed to address the imbalance in our classification dataset, which comprises Appropriate for Gestational Age (AGA) (82.99%), LGA (11.67%), and SGA (5.34%) infants. SMOTE, a widely used oversampling method, selects examples close to the feature space, constructs a line between these examples, and generates a new sample at a point along this line. Furthermore, an undersampling technique is also implemented on our dataset, as recommended by the authors [31]. This combination of oversampling and undersampling techniques aids in enhancing the robustness of the classification model.

#### 3.1.3. Feature Selection

The computation and determination of feature importance are crucial in this phase, identifying the most pertinent features that best represent our dataset and contribute to optimal classification accuracy. We combine expert-driven feature selection to remove irrelevant features with a proposed feature selection algorithm to evaluate a classifier and its generated feature importance list, addressing the drawbacks of automatic feature selection approaches in machine learning. This multifaceted approach enhances the model’s performance by retaining only the most relevant features, fostering robust and accurate predictions.

First, our research aims to identify factors associated with SGA-LGA at the end of the first trimester. Therefore, we exclude irrelevant features from the full feature set. This process results in a subset termed *ManualFeature*, which excludes features such as postpregnancy weight, postpartum bleeding, postpartum bleeding volume, birth weight, week of gestational delivery, preterm birth, and low Apgar scores (1–7 min).

Second, we propose a feature selection algorithm, detailed in pseudocode of Algorithm 1. This algorithm requires the input classifier to support the calculation of feature importance scores or coefficients. If our proposed classifier (Algorithm 2) is used in Algorithm 1, these scores are calculated as in Section 3.1.5. The calculation steps are as follows:

- In Step 1, the feature importance scores are computed, converted to absolute values, and sorted in descending order. A table named *results* is initialized to store the outcomes.
- In Step 2, each score is treated as a threshold for selecting features and the corresponding data with importance scores greater than or equal to this threshold. Subsequently, *k*-fold cross-validation with a stratified strategy is applied across *n* iterations to train the classifier. The mean accuracy, standard deviation, and accuracy change (delta accuracy) are then computed from the validation fold and saved to the results table, along with the selected features, for later comparison.
- In Step 3, the results table is sorted in descending order by mean accuracy and in ascending order by standard deviation. The optimal feature group is selected based on the highest mean accuracy (or close to it), smaller standard deviation of accuracy, and smaller number of features. This group is identified as the last element of the first row of the sorted table.

**Algorithm 1:** Find the best feature group.
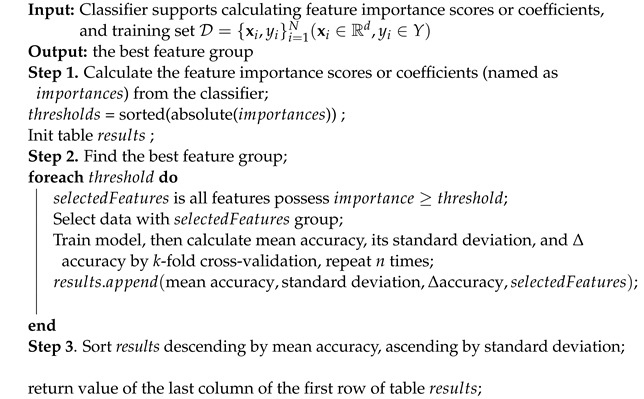


    The main idea of our feature selection algorithm is that as each feature is added from the descending sorted list of absolute feature importance scores, the classifier’s performance will increase and reach a peak if the list and the classifier are appropriate. It will then achieve stability with the remaining features in the list. Our proposed feature selection algorithm can evaluate both the feature importance list and the classifier.

**Figure 2:**
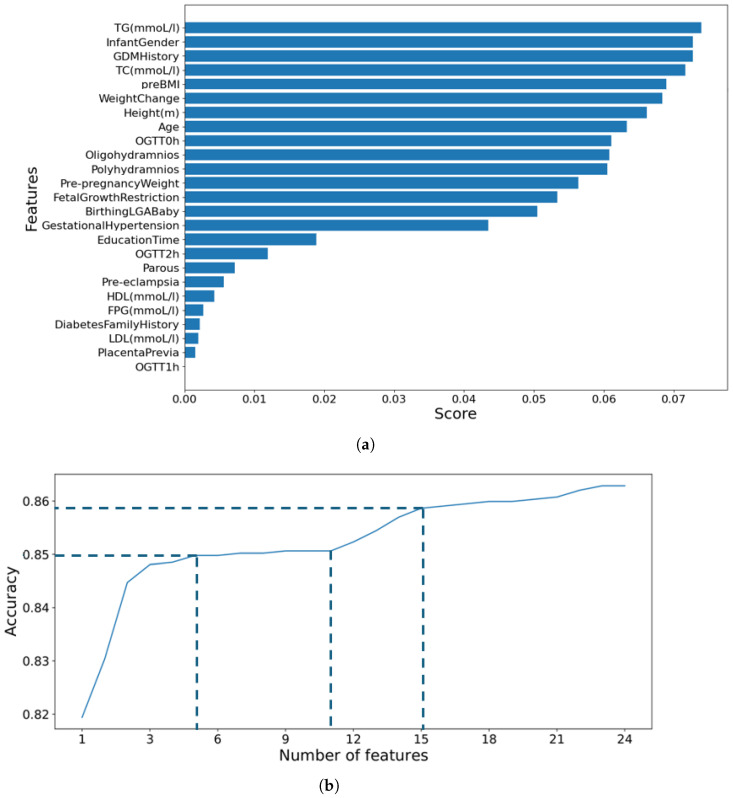
Illustration of the disadvantages of automatic feature selection approaches in ML. (**a**) Feature importance list generated by Random Forest classifier. (**b**) Run Algorithm 1 with Random Forest classifier to find the best feature group.

To illustrate the disadvantages of conventional automatic feature selection approaches in machine learning, which typically select the top *k* features based on a feature importance list, we use the Random Forest (RF) classifier to generate feature importance scores for the *ManualFeature* group, as shown in Figure 2a. We then run Algorithm 1 with the RF classifier, and the result is presented in Figure 2b. From Figure 2, it is evident that the top five features, from *TG* to *preBMI*, contribute approximately 85.0% to the RF’s performance accuracy. Despite having high importance scores, the next six features, from *WeightChange* to *Polyhydramnios*, do not significantly enhance the RF’s performance. This indicates that these features, generated by the RF classifier, are inappropriate for inclusion in the list. The subsequent four features, from *Prepregnancy Weight* to *Gestational Hypertension*, add approximately 1.0% (86%−85%) to the accuracy, and the remaining features contribute only about 0.4% to the accuracy.

In conclusion, if the feature importance score list and the classifier that generated this list are accurate, adding features in order from the highest to the lowest score should enhance the model’s performance and achieve stability. Conversely, if the feature importance list and classifier are inaccurate, this improvement will not occur, as illustrated in Figure 2. Algorithm 1 can assess the accuracy of the feature importance list and determine whether the classifier is appropriate. If the classifier is suitable, Algorithm 1 can identify the optimal feature group corresponding to the highest or near-highest mean accuracy and the narrowest standard deviation. This ensures the classifier remains stable even when additional, less important features are included. Further details on the results of feature selection using our proposed classifier are provided in Section 4.2.

We execute Algorithm 1 using our proposed classifier (Algorithm 2) as the input classifier to validate our proposed classifier and identify the best feature group representative of our dataset. The results are presented in Section 4.2.
**Algorithm 2:** Propose classifier for SGA-LGA dataset.
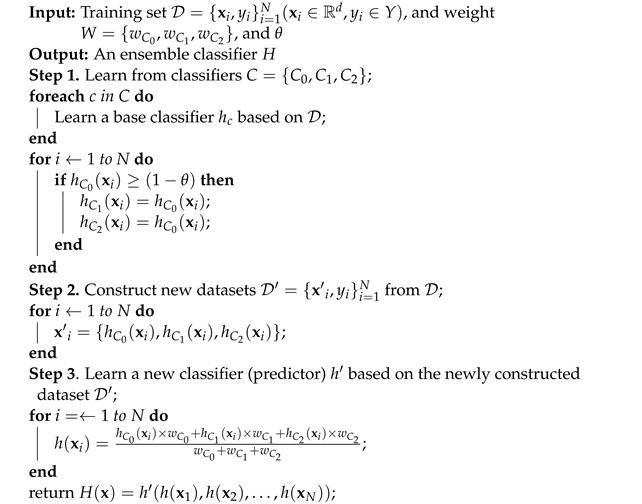


#### 3.1.4. Selection of the Most Effective Algorithm

**Figure 3:**
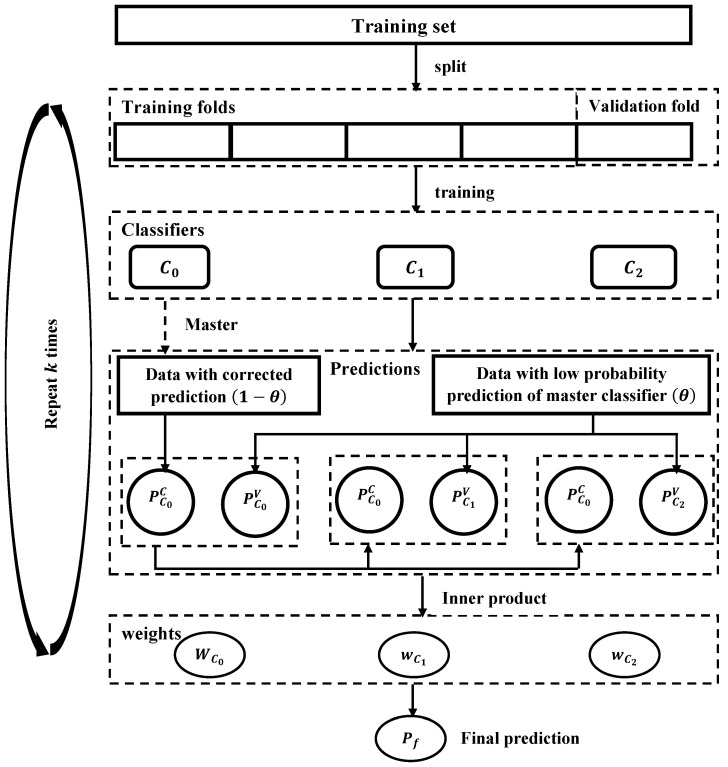
Our proposed classifier for SGA-LGA data.

The algorithm proposed for classifying SGA, LGA, and AGA fetuses is illustrated in Figure 3. The process involves the construction of new data based on the classifiers’ predictions. Three distinct prediction metrics, namely $P_{C_{0}}$, $P_{C_{1}}$, and $P_{C_{2}}$, are associated with the primary classifier $C_{0}$, and two secondary classifiers $C_{1}$ and $C_{2}$, respectively. Each prediction metric is divided into corrected (or inherited from the primary classifier $C_{0}$) and voting metrics. This results in the decomposition of $P_{C_{0}}$ into $P_{C_{0}}^{C}$ and $P_{C_{0}}^{V}$, $P_{C_{1}}$ into $P_{C_{1}}^{C}$ and $P_{C_{1}}^{V}$, and $P_{C_{2}}$ into $P_{C_{2}}^{C}$ and $P_{C_{2}}^{V}$. The corrected or inherited part is specifically determined by the primary classifier ($C_{0}$), denoted as $P_{C_{0}}^{C}$, from data with corrected predictions represented by 1−θ (where θ signifies a low or uncertain probability prediction). Subsequently, $P_{C_{1}}^{C}$ and $P_{C_{2}}^{C}$ are replaced (or inherited) by $P_{C_{0}}^{C}$, i.e., $P_{C_{0}}^{C}\equiv P_{C_{1}}^{C}\equiv P_{C_{2}}^{C}$. The voting metrics, including $P_{C_{0}}^{V}$, $P_{C_{1}}^{V}$, and $P_{C_{2}}^{V}$, are utilized to classify using a weighted voting mechanism with the weight matrix $W=\{w_{C_{0}},w_{C_{1}},w_{C_{2}}\}$, ultimately resulting in the creation of a new predictor. Further details about this proposed classifier are elucidated through pseudocode in Algorithm 2.

In this phase, we standardize the dataset and then split the data into a training set (75%) and a testing set (25%) using a stratified strategy. Five state-of-the-art machine learning algorithms are scrutinized, including (1) RF, (2) k-Nearest Neighbors (KNNs), (3) CART, (4) SVM, and (5) XGB. Each algorithm was fine-tuned to optimize performance on the same dataset. The optimal hyperparameters for each algorithm are as follows:

1. RF’s parameters: criterion = ‘log_loss’, max_depth = 13;
2. KNN’s parameters: leaf_size = 5, n_neighbors = 24, p = 1, weights = ‘distance’;
3. CART’s parameter: criterion = ‘entropy’;
4. SVM’s parameters: C = 100, cache_size = 1000, decision_function_shape = ‘ovo’, degree = 2, probability = True;
5. XGB’s parameters: booster = ‘gbtree’, colsample_bytree = 0.5, learning_rate = 0.01, colsample_bylevel = 0.6, max_depth = 4, min_child_weight = 2, missing = nan, n_estimators = 1000, num_class = 3.

**Figure 4:**
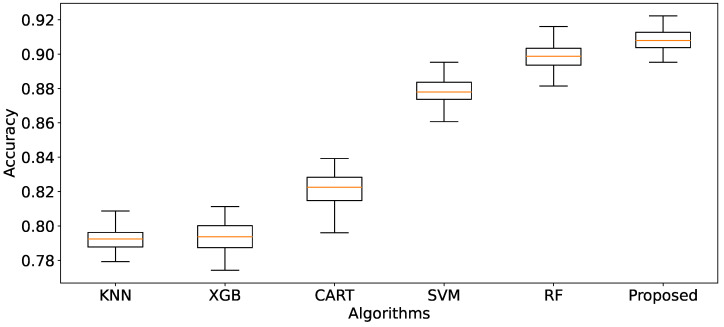
Comparison of algorithms using the *ManualFeature* group, evaluated by mean accuracy across 100 iterations of 10-fold cross-validation.

From Figure 4, the ranking of the five state-of-the-art algorithms in terms of mean accuracy is RF, SVM, CART, XGB, and KNN. To identify three classifiers and the weight metric *W* for our proposed classifier, we designate the primary classifier, either RF or SVM (RF is the best among ensemble tree algorithms—RF, CART, XGB—and SVM, ranked second, is a different type of algorithm). We then select two secondary classifiers from the remaining algorithms (RF/SVM (whichever differs from the primary classifier), CART, XGB, and KNN) by executing Algorithm 3. This algorithm allows us to determine the optimal classifiers, $W=\{w_{C_{0}},w_{C_{1}},w_{C_{2}}\}$, and θ. The optimal parameters are as follows: the primary classifier is *RF*, the two secondary classifiers are *XGB* and *SVM*, and the parameter set $\left(\theta ,w_{RF},w_{XGB},w_{SVM}\right)=\left(0.001,0.19,0.01,0.73\right)$. These parameters will be applied to other relevant algorithms for execution. The proposed solution is then implemented and compared to identify the most effective approach.

Subsequently, identifying a subset of features representing the entire dataset is performed. To achieve this, fine-tuning of all algorithms is conducted to ascertain the optimal parameters, ensuring the highest accuracy for each algorithm. Then, implementation of 10-fold cross-validation with the training set and iterate the process 100 times to assess the performance of six distinct approaches for data classification. The best accuracy (Acc) and Mean Squared Errors (MSE) of the fine-tuned algorithms are compared, aiding in selecting the most robust model. This selected model is then used to determine the best feature group described in Section 3.1.3, which is used in the subsequent step to find the feature importance within the dataset.
**Algorithm 3:** Fine-tuning θ and classifiers’ weights for Algorithm 2.
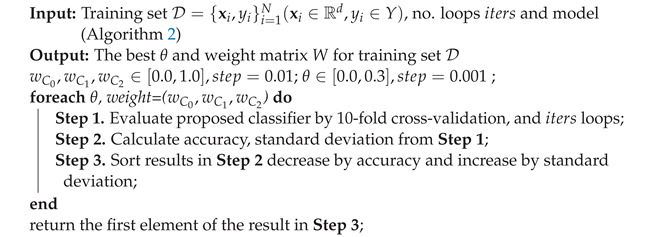


#### 3.1.5. Determination of Feature Importance

Assume that we need to classify data $D={\left\{x_{n},y_{n}\right\}}_{n=1}^{N}\left(x_{n}\in R^{d},y_{n}\in Y\right)$ into three classes (N=3): SGA, AGA, and LGA. From Figure 3, the determination of feature importance is proposed to be achieved through a two-phase analysis: (1) analysis of classifiers and (2) proposal of a new predictor. For phase (1), three classifiers are selected: RF, XGB, and SVM. The feature importance scores (or coefficients) are calculated for each classifier. The detailed methods are presented below:

Extreme Gradient Boost (XGBoost or XGB) is an ensemble learning method that uses tree ensembles as base learners, with the trees belonging to the CART family. In XGB, the ensemble model comprises a set of these trees constructed sequentially, where each subsequent iteration aims to reduce misclassification rates. Assuming we have *K* trees, the model predicts the *n*th training data, $x_{n}$, as ${\hat{y}}_{n}=\sum_{k=1}^{K}f_{k}\left(x_{n}\right)$,$f_{k}\in F$ where *F* represents the function space containing all regression trees [11]. XGB focuses on creating learning functions (trees) that store scores in their leaves. XGB employs a boosting technique consisting of three steps:

1. Defining an initial model ${\hat{y}}_{n}^{\left(0\right)}$ to predict the target variable $y_{n}$, with this model associated with a residual $\left(y_{n}-f_{0}\left(x_{n}\right)\right)$;
2. Fitting a new model $f_{1}\left(x_{n}\right)$ to the residuals from the previous step;
3. Combining ${\hat{y}}_{n}^{\left(0\right)}$ and $f_{1}\left(x_{n}\right)$ to obtain ${\hat{y}}_{n}^{\left(1\right)}$, the boosted version of ${\hat{y}}_{n}^{\left(0\right)}$.

This process can be iterated for *t* rounds, resulting in ${\hat{y}}_{n}^{\left(t\right)}$ until residuals are minimized. The objective at round *t* is expressed as $Obj^{\left(t\right)}=\sum_{n=1}^{N}l\left(y_{n},{\hat{y}}_{n}^{\left(t-1\right)}+f_{t}\left(x_{n}\right)\right)+\Omega \left(f_{t}\right)+\mathrm{constant}$, where the goal is to find $f_{t}$ to minimize this objective. To enhance readability, we use the notation $XGB=[xgb_{1},xgb_{2},\ldots ,xgb_{d}]$ to represent the scores of attributes instead of $\left[f_{1},f_{2},\ldots ,f_{d}\right]$ [11].

Support Vector Machine (SVM) uses a hyperplane and maximal margin between classes (binary classification). Let us build N(N−1)=3×(3−1)=6 classifiers where each classifier distinguishes each pair of classes *i* and *j*. And denote $f_{ij}\left(x\right)=w_{ij}^{T}x_{ij}+b_{i}$ is a classifier where class *i* is all positive samples, and the rest belongs to class *j*. Our problem is equal to: $\left(w_{i},b_{i}\right)=\arg\max_{\left(w_{i},b_{i}\right)}\left(w_{i}^{T}x_{i}+b_{i}\right)$. Solve this equation to obtain coefficients w and intercept *b* of SVM. They are feature importance scores. Conveniently, w will replaced by $SVM=[svm_{1},svm_{2},\ldots ,svm_{d}]$.

Random Forest (RF) is a popular machine learning algorithm that belongs to the ensemble learning method. It operates by constructing many decision trees at training time and outputting the class, that is, the mode of the classes (classification) or mean prediction (regression) of the individual trees.

Random Forests are corrected for decision trees’ habit of overfitting their training set. The fundamental concept behind RF is to combine the predictions made by many decision trees into a single model. Individually, decision trees’ predictions may not be accurate, but combined, they can produce accurate and stable predictions.

The goal of RF algorithm is to construct a function f:D→Y such that the error $\sum_{i}^{N}{\left|f_{i}\left(x\right)-y_{i}\right|}^{2}$ is minimized. While constructing trees by adding nodes, we calculate the importance of the features. The importance of a feature is computed as the total reduction in the criterion brought by that feature. It is also known as the Gini importance. It is a measure of the contribution of each feature to the overall reduction in impurity (Gini index) achieved by the decision tree. It is calculated as follows:

$$\mathrm{Gini}\;\mathrm{Importance}=1-\sum_{i=1}^{3}{\left(p_{i}\right)}^{2}-\frac{N_{\mathrm{left}}}{N_{\mathrm{parent}}}\times {\mathrm{Gini}}_{\mathrm{left}}+\frac{N_{\mathrm{right}}}{N_{\mathrm{parent}}}\times {\mathrm{Gini}}_{\mathrm{right}}$$

where $p_{i}$ is the proportion of instances belonging to class *i* in the node (before splitting), and $N_{\mathrm{left}}$ and $N_{\mathrm{right}}$ are the number of instances in the left and right child nodes, respectively. $Gini_{left}$ and $Gini_{right}$ are Gini left and right of the parent node.

The Gini importance provides a way to rank features based on their contribution to the decision tree’s ability to make accurate predictions. Features with higher Gini importance are considered more influential in the decision-making process of the tree. It’s worth noting that this approach is specific to decision trees, and other machine learning algorithms may have different methods for evaluating feature importance. We denote RF features importance as $RF=\{{\mathrm{rf}}_{1},{\mathrm{rf}}_{2},\ldots ,{\mathrm{rf}}_{d}\}$ for convenience.

According to the proposed classifier presented in Algorithm 2, as a result of phase 1, three sets of feature importance scores (or coefficients) are generated for each classifier (XGB, SVM, and RF). In phase 2, to propose the new predictor, a set of weights $\left\{w_{XGB},w_{SVM},w_{RF}\right\}$ is used for each corresponding classifier, respectively. Hence, the new feature importance (denoted as fi) is calculated as follows:

$$fi_{i}=\frac{|xgb_{i}|\times w_{XGB}+|svm_{i}|\times w_{SVM}+\left|rf_{i}\right|\times w_{RF}}{w_{XGB}+w_{SVM}+w_{LR}}$$

with $i=\bar{1..d}$.

The proposed calculation steps are explained as pseudocode in Algorithm 4:
**Algorithm 4:** Find feature importance scores based on our proposed classifier.
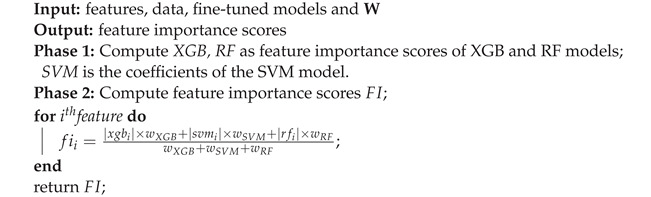


## 4. Results

### 4.1. Model Selection

We compared the average accuracies and Mean Squared Errors (MSEs) of five state-of-the-art algorithms using *ManualFeature* group with our proposed algorithm. Each algorithm has been fine-tuned on *ManualFeature* to obtain the best parameters. From Figure 4, our proposed solution is outstanding in mean accuracy (90.80%), and the standard deviation of accuracy is the narrowest. Then, two good models, RF and SVM, achieve mean accuracy of 89.85% and 87.81%, respectively. The other models achieve mean accuracies from 79.22% to 82.17%. It means our proposal algorithm achieves the highest accuracy and is more stable than others.

**Figure 5:**
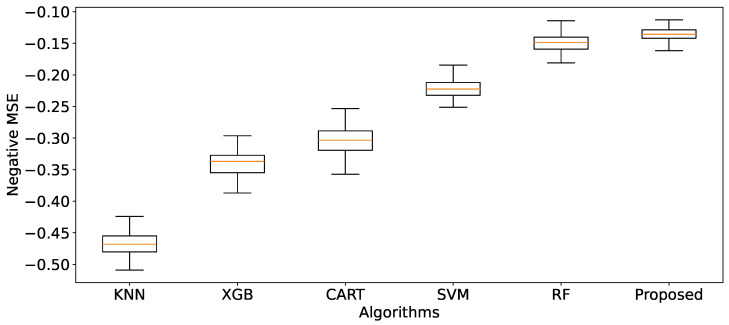
Comparison of algorithms using the *ManualFeature* group, evaluated by negative MSE across 100 iterations of 10-fold cross-validation.

Furthermore, the negative MSE results are also presented in Figure 5. They show that the error rate for the proposed solution is the smallest MSE (synonym with the biggest negative MSE) and narrowest compared with other machine learning algorithms. Next, in order, are RF, SVM, CART, and XGB; the last is KNN. In other words, the proposed solution has a better prediction performance.

In conclusion, the proposed solution outperforms other state-of-the-art algorithms regarding prediction accuracy, stability, and error minimization.

### 4.2. Feature Selection

**Figure 6:**
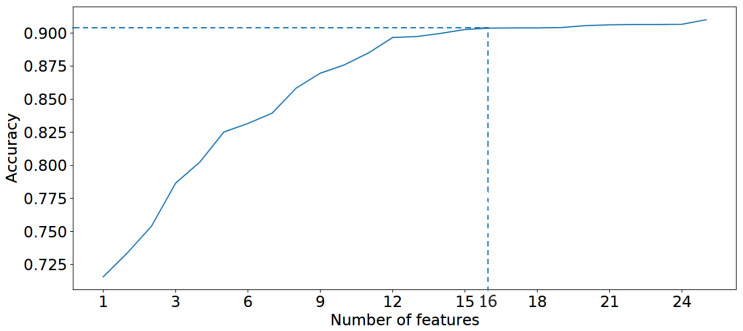
Find the best feature group based on Algorithm 1.

**Figure 7:**
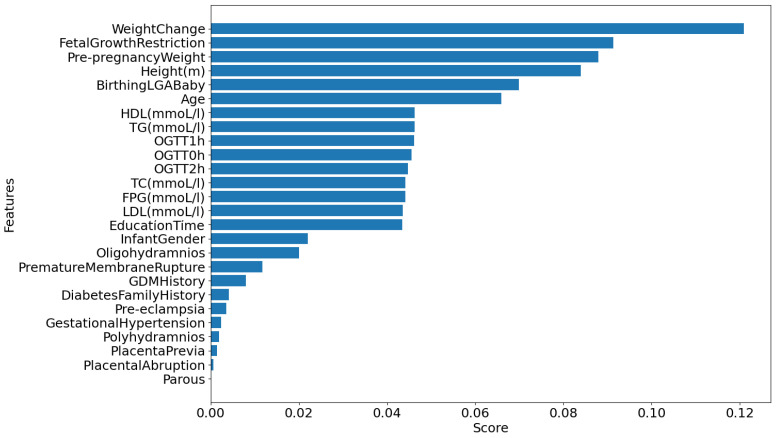
Feature importance list generated by our proposed classifier on *ManualFeature* group.

After identifying the best model (in this case, our proposed model), we executed Algorithm 1 using the *ManualFeature* group to determine the optimal feature group. Figure 6 presents the result of finding the best feature group by executing Algorithm 1 with the *ManualFeature* group and our proposed classifier. As explained in Section 3.1.3, the performance of our proposed classifier increases from the *weight change* feature to the *infant gender* feature, reaches a peak, and then stabilizes. This indicates that the classifier achieved its highest accuracy with 16 out of 24 features in the feature importance list (presented in Figure 7) generated by our proposed classifier. This optimal feature group, named the *BestFeature* group, consists of *weight change, fetal growth restriction, prepregnancy weight, height, age, birthing LGA baby, HDL, TG, OGTT-1h, OGTT-0h, OGTT-2h, TC, FPG, LDL, education time, and infant gender*.

We rerun five selected algorithms and our proposed algorithm using the *BestFeature* group, evaluated by mean accuracy and negative MSE across 100 iterations of 10-fold cross-validation. We combine these results with the previously reported outcomes for the *ManualFeature* group, as presented in Table 4. It shows the proposed algorithm achieves the highest accuracy again, about 92.12%, higher before around 1.32%, and narrower standard deviation than before (0.0059 vs. 0.0066), while others stand lower. Almost all models achieve higher accuracy and smaller standard deviation; only CART and XGB get slightly lower accuracy (0.8215 vs. 0.8217 and 0.7794 vs. 0.7939).

Furthermore, Table 4 shows that our model’s negative MSE is approximately −0.1197, an improvement over the previous value of −0.1356 evaluated in the *ManualFeature* group. Additionally, our proposed algorithm demonstrates the lowest standard deviation in MSE, recorded at 0.0117, compared with the earlier value of 0.0120. In contrast, other models exhibit higher MSEs and lower standard deviations, except for the XGB and CART models.

In conclusion, our proposed model is the most outperforming, and the *BestFeature* group represents our dataset’s most suitable feature group.

Suppose we choose a testing size = 0.25 with a random partition. In this case, we have a confusion matrix and classification report of *BestFeature*, as in Table 5. The average accuracy is 92%; besides that, the precision, recall, and F1-score of the SGA classification are the most balanced and achieve the highest scores, corresponding to 0.95,0.96, and 0.95, respectively, while the values of the LGA classification correspond to 0.85,0.95, and 0.90. Furthermore, the scores of the AGA classification are 0.96,0.85, and 0.90. Overall, these values are high and balanced in classification.

### 4.3. Find Feature Importance

**Figure 8:**
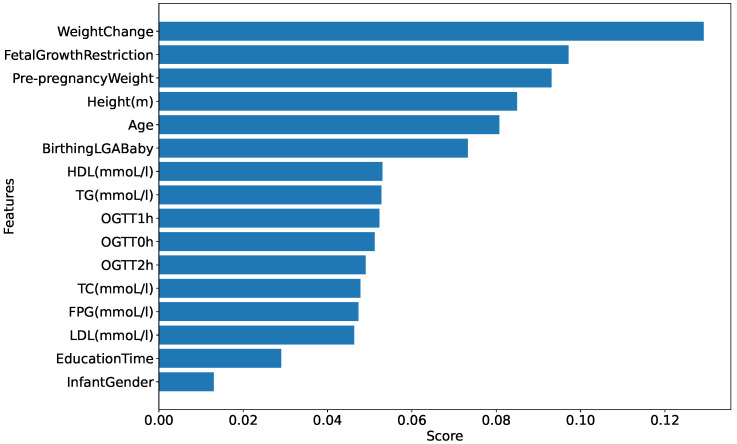
Feature importance generated by Algorithm 4 on *BestFeature* group.

We use Algorithm 4 to determine the feature importance for the SGA-LGA dataset based on the *BestFeature* group. The results are presented in Figure 8. Additionally, we employ the SHAP (SHapley Additive exPlanations) beeswarm plot in our feature importance analysis. This plot, a powerful visualization tool, helps interpret the output of machine learning models by displaying the impact of each feature on the model’s predictions. This allows for a detailed understanding of feature importance and interaction [32].

**Figure 9:**
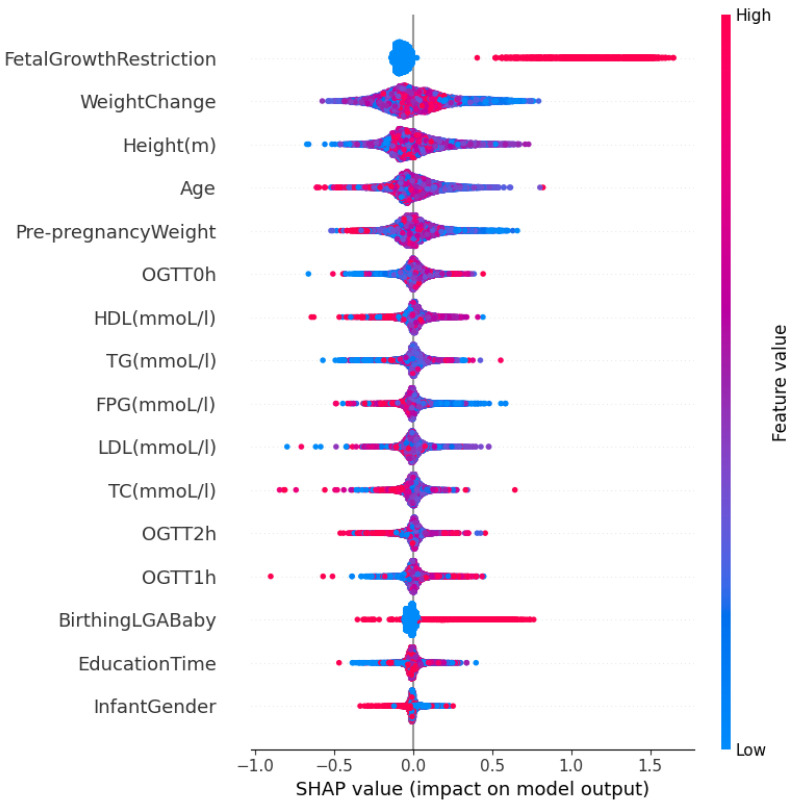
Beeswarm plot of feature importance based on SHAP values using *BestFeature* group.

Figure 9 presents a beeswarm plot of feature importance for our *BestFeature* group based on SHAP values. This figure indicates that fetal growth restriction is a high-risk factor leading to SGA outcomes, while birthing LGA baby is more likely to result in LGA outcomes. Other features in the list also exhibit mixed and unclear effects on the classification of SGA, LGA, and AGA. Moreover, the order of feature importance in this figure differs from that in Figure 8, which is proved correctly as discussed in Section 4.2 above.

For a deeper understanding of the feature importance list, we divide features into groups based on the effect or change in importance of each feature in order of feature importance score:

1. Maternal and Obstetric Factors: In order, the features with the highest feature importance score are weight change, fetal growth restriction, prepregnancy weight, height, age, and birthing LGA baby. Referring to Figure 6, this feature group is estimated to achieve an overall accuracy of approximately 82.5% within the BestFeature group, indicating their significant relevance to the SGA or LGA outcomes. To elucidate further, we reorganize this group into two categories: Maternal Factors (weight change, prepregnancy weight, height, age) and Obstetric Factors (fetal growth restriction, birthing LGA baby). The rationale behind this classification is that maternal factors are likely to influence the SGA or LGA outcomes. In contrast, obstetric factors such as fetal growth restriction or a history of birthing LGA baby may exacerbate the likelihood of subsequent SGA or LGA infants.
2. First Trimester Biomarker Factors: They are medium feature importance scores. In descending order, this group comprises the following features HDL, TG, OGTT-1h, OGTT-0h, OGTT-2h, TC, FPG, and LDL. Thus, this group is estimated to contribute approximately 7.2% (89.7–82.5%) (more details in Figure 6) to the classification performance, enhancing diagnostic accuracy by providing additional factors.
3. Other Factors: Including other features, such as education time and infant gender, improves the model’s accuracy by approximately 2.4% (92.1–89.7%) marginally.

## 5. Discussion

Exploring clinical demographics and biomarkers at the first trimester ending for fetuses with SGA-LGA conditions presents a promising avenue for research. The proposed system aims to aid medical practitioners and researchers identify critical features from SGA-LGA data essential for clinical practice.

As presented in the results, the system highly recommends clinical experts focus on the most significant maternal and obstetric attributes are weight change, prepregnancy weight, height, age and fetal growth restriction, birthing LGA baby, respectively. It also emphasizes the importance of double-checking biomarker features at the end of the first trimester, including HDL, TG, OGTT-1h, OGTT-0h, OGTT-2h, TC, FPG, and LDL. These features of importance are illustrated in Figure 8.

From Figure 8, inadequate weight gain in early pregnancy is associated with a higher risk of SGA, while excessive weight gain increases the likelihood of LGA [33,34]. A higher prepregnancy BMI (calculated from weight and height) correlates with LGA, whereas a lower BMI is linked to SGA. Short maternal stature is a risk factor for SGA, while taller mothers have a higher likelihood of LGA [33]. Advanced maternal age raises the risk of both SGA and LGA due to complications such as gestational diabetes and hypertension [34,35]. These findings underscore the importance of monitoring maternal characteristics such as weight, height, and age during prenatal care to reduce the risks associated with abnormal fetal growth [34,35].

Additionally, biomarkers measured at the end of the first trimester reflect the status of SGA or LGA fetuses. Elevated OGTT-1h and OGTT-2h levels are linked to increased risks of gestational diabetes and LGA, while lower levels may be associated with SGA due to insufficient glucose supply. Similarly, elevated fasting plasma glucose (FPG) in the first trimester predicts a higher risk of LGA due to persistent maternal hyperglycemia, whereas lower FPG levels can be a risk factor for SGA [36,37]. Higher triglyceride (TG) levels are related to LGA risk, whereas lower high-density lipoprotein (HDL) levels are associated with SGA, indicating the importance of lipid balance for optimal fetal growth [37]. These findings emphasize the need for early monitoring and management of maternal metabolic health to improve pregnancy outcomes.

The human biological system is complex and dynamic: a small change initially can lead to significant changes later in life. Although our findings highlight high-risk factors that can support clinical practitioners in assessing and then applying timely and suitable interventions, the effectiveness of such interventions remains uncertain. There is a lack of research quantifying the effect of timely, completely isolated interventions on these high-risk factors for SGA and LGA. We highly recommend further research to quantify the effect of timely interventions for SGA-LGA. As discussed in our study, many risk factors for SGA-LGA affect fetuses, infants, mothers, and their childhood development. Quantifying the impact of interventions and applying proven effective measures can result in significant positive changes in the outcomes for mothers, fetuses, and children.

In terms of performance, the proposed solution has demonstrated superior accuracy and lower MSE levels compared with five contemporary algorithms on both the *ManualFeature* and *BestFeature* groups, as depicted in Table 4.

Our proposed Algorithm 1 seeks to identify the optimal feature group by selecting the feature importance threshold. It then returns the most suitable feature group with the highest or near-highest accuracy and the smallest standard deviation of accuracy. This approach proposes reducing the number of analysis features from 26 to 16, thereby enhancing model performance.

Concerning the classification system, the proposed solution achieved an average accuracy of 0.92. Each classification class—SGA, LGA, and AGA—demonstrated high and balanced precision, recall, and F1-score values, as presented in Table 5. These results suggest that our proposed solution surpasses others and will likely yield accurate predictions on additional validation sets.

## 6. Conclusions

In conclusion, classifying SGA and LGA fetuses is essential for identifying at-risk groups and developing targeted clinical management strategies. Effective interventions for SGA include close monitoring, dietary adjustments, and maternal nutritional support, while LGA management requires strict glycemic control and careful obstetric oversight to prevent birth complications.

This study introduces a computational system that classifies fetuses as SGA, LGA, and AGA by the end of the first trimester using maternal demographics, factors, and biomedical parameters collected between 10 to 13 weeks of pregnancy. The system also identifies key classification features to enhance computational and clinical analysis.

The proposed model demonstrated high accuracy, with an average of 92.12% through 10-fold cross-validation over 100 loops, and outperformed five advanced machine learning algorithms in accuracy and MSE. It achieved 92% accuracy on the test set, showing high and balanced precision, recall, and F1-score for SGA, LGA, and AGA classifications, as detailed in Table 5.

Additionally, the system selected relevant attributes for medical analysis, such as maternal factors (weight change, prepregnancy weight, height, age) and obstetric factors (fetal growth restriction, birthing LGA baby), and certain biomarkers at the end of the first trimester, including HDL, TG, OGTT-1h, OGTT-0h, OGTT-2h, TC, FPG, LDL, among others. These were determined based on feature importance.

Future research may investigate the application of artificial intelligence techniques, including recurrent neural networks (RNNs) and Convolutional Neural Networks (CNNs), to enhance the predictive capabilities for classification tasks.

## Figures and Tables

**Table 1:** SGA-LGA implications for mothers, fetuses, and in childhood [3,4,7,8,9,10,11].

**SGA Implications**	**Detail**
Risk during pregnancy	pre-eclampsia
Risk of neonatal complication	respiratory distress syndrome, hypoglycemia, hypothermia
Infection	weakened immune system, more susceptible to infections
Developmental delays	motor skills, cognitive development, language development
Long-term growth issues	shorter stature, lower weight
Chronic diseases	type 2 diabetes, cardiovascular disease, hypertension
Cognitive and behavioral challenges	learning disabilities, attention deficit hyperactivity disorder
Nutritional challenges	special nutritional support; growth, development monitoring
Delayed puberty	social, psychological
Psychosocial and emotional impacts	
**LGA Implications**	**Detail**
Risk during pregnancy	pre-eclampsia
Risk gestational diabetes	
Birth complications	shoulder dystocia, injuries for infant; injuries for mother
Cesarean section	
Macrosomia	
Neonatal hypoglycemia	
Respiratory distress syndrome	
Long-term health risks	obesity, type 2 diabetes, cardiovascular
Metabolic issues	metabolic syndrome, type 2 diabetes
Developmental delays	developmental delays, cognitive challenges
Childhood obesity	
Psychosocial and emotional impacts	

**Table 2:** SGA-LGA attributes by group.

Group	Name
Common maternal attributes	age, height, prepregnancy weight
Maternal history attributes	education time, diabetes family history, GDM history, birthing LGA baby, parous
Current maternal attributes	postpregnancy weight, weight change, pre-BMI, premature membrane rupture, placental abruption, gestational hypertension, pre-eclampsia, placenta previa, polyhydramnios, oligohydramnios, postpartum bleeding volume, postpartum bleeding, FPG, TC, TG, HDL, LDL, TyG index, OGTT-0h, OGTT-1h, OGTT-2h
Fetal attributes	gender, fetal growth restriction
Infant attributes	low Apgar (1–7 min), birth weight, preterm birth, week of gestational delivery, LGA, SGA

**Table 3:** Data brief after removing unnecessary columns and duplicate/invalid rows.

Columns/Features/Attributes	No. Missing Values
Gestational Hypertension	1
Pre-eclampsia	3
Postpartum Bleeding Volume	4
FPG	15
TC	1065
TG	1073
HDL, LDL	1079
OGTT-0h	39
OGTT-1h	44
OGTT-2h	46
1133 rows/samples with missing values	4448 missing values

**Table 4:** Comparison of algorithms performance: accuracy and negative MSE for *ManualFeature* versus *BestFeature* groups.

Group	Model	Mean Acc	Stddev	Negative MSE	Stddev
ManualFeature	KNN	0.7922	0.0068	−0.4675	0.0182
	XGB	0.7939	0.0089	−0.3404	0.0215
	CART	0.8217	0.0091	−0.3040	0.0234
	SVM	0.8781	0.0071	−0.2226	0.0161
	RF	0.8985	0.0078	−0.1489	0.0154
	Proposed	0.9080	0.0066	−0.1356	0.0120
BestFeature	KNN	0.7961	0.0056	−0.4556	0.0173
	XGB	0.7794	0.0100	−0.3623	0.0197
	CART	0.8215	0.0102	−0.3138	0.0280
	SVM	0.8478	0.0070	−0.1197	0.0117
	RF	0.9131	0.0064	−0.1323	0.0127
	Proposed	0.9212	0.0059	−0.1197	0.0117

**Table 5:** Confusion matrix and classification report of our proposed classifier on *BestFeature* group.

Confusion Matrix		Precision	Recall	F1-Score	Support
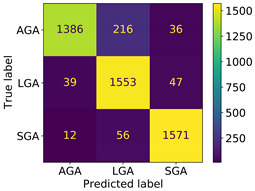	AGA	0.96	0.85	0.90	1638
	LGA	0.85	0.95	0.90	1639
	SGA	0.95	0.96	0.95	1639
	accuracy			0.92	4916
	macro avg	0.92	0.92	0.92	4916
	weighted avg	0.92	0.92	0.92	4916
				
				
				
				
				

## Data Availability

Data is unavailable due to privacy or ethical restrictions.

## References

[1] Crispi F., Crovetto F., Gratacos E. (2018). Intrauterine growth restriction and later cardiovascular function. Early Hum. Dev..

[2] Black R.E., Victora C.G., Walker S.P., Bhutta Z.A., Christian P., De Onis M., Ezzati M., Grantham-McGregor S., Katz J., Martorell R. (2013). Maternal and child undernutrition and overweight in low-income and middle-income countries. Lancet.

[3] Ananth C.V., Ananth C.V., Vintzileos A.M. (2006). Epidemiology of preterm birth and its clinical subtypes. J. Matern.-Fetal Neonatal Med..

[4] Arias F., Bhide A.G., Arulkumaran S., Damania K., Daftary S.N. (2008). Practical Guide to High Risk Pregnancy and Delivery-E-Book: A South Asian Perspective.

[5] Morkuniene R., Cole T.J., Jakimaviciene E.M., Bankauskiene A., Isakova J., Drazdiene N., Basys V., Tutkuviene J. (2023). Regional references vs. international standards for assessing weight and length by gestational age in Lithuanian neonates. Front. Pediatr..

[6] Erchick D.J., Hazel E., Katz J., Lee A., Diaz M., Wu L., Yoshida S., Bahl R., Grandi C., Labrique A. (2023). Vulnerable newborn types: Analysis of subnational, population-based birth cohorts for 541 285 live births in 23 countries, 2000–2021. BJOG Int. J. Obstet. Gynaecol..

[7] Catalano P.M., Shankar K. (2017). Obesity and pregnancy: Mechanisms of short term and long term adverse consequences for mother and child. BMJ.

[8] Pedersen J. (1977). The Pregnant Diabetic and Her Newborn: Problems and Management.

[9] Broskey N.T., Wang P., Li N., Leng J., Li W., Wang L., Gilmore L.A., Hu G., Redman L.M. (2017). Early pregnancy weight gain exerts the strongest effect on birth weight, posing a critical time to prevent childhood obesity. Obesity.

[10] Manandhar T., Prashad B., Nath Pal M. (2018). Risk factors for intrauterine growth restriction and its neonatal outcome. Gynecol Obs..

[11] Nguyen Van S., Lobo Marques J., Biala T., Li Y. (2021). Identification of Latent Risk Clinical Attributes for Children Born Under IUGR Condition Using Machine Learning Techniques. Comput. Methods Programs Biomed..

[12] Chen Q., Wei J., Tong M., Yu L., Lee A., Gao Y., Zhao M. (2015). Associations between body mass index and maternal weight gain on the delivery of LGA infants in Chinese women with gestational diabetes mellitus. J. Diabetes Its Complicat..

[13] Shen Y., Zhao W., Lin J., Liu F. (2017). Accuracy of sonographic fetal weight estimation prior to delivery in a Chinese han population. J. Clin. Ultrasound.

[14] Blue N.R., Yordan J.M.P., Holbrook B.D., Nirgudkar P.A., Mozurkewich E.L. (2017). Abdominal circumference alone versus estimated fetal weight after 24 weeks to predict small or large for gestational age at birth: A meta-analysis. Am. J. Perinatol..

[15] Feng M., Wan L., Li Z., Qing L., Qi X. (2019). Fetal weight estimation via ultrasound using machine learning. IEEE Access.

[16] Tao J., Yuan Z., Sun L., Yu K., Zhang Z. (2021). Fetal birthweight prediction with measured data by a temporal machine learning method. BMC Med. Inform. Decis. Mak..

[17] Mobadersany P., Cooper L.A., Goldstein J.A. (2021). GestAltNet: Aggregation and attention to improve deep learning of gestational age from placental whole-slide images. Lab. Investig..

[18] Khan W., Zaki N., Masud M.M., Ahmad A., Ali L., Ali N., Ahmed L.A. (2022). Infant birth weight estimation and low birth weight classification in United Arab Emirates using machine learning algorithms. Sci. Rep..

[19] Lee L.H., Bradburn E., Craik R., Yaqub M., Norris S.A., Ismail L.C., Ohuma E.O., Barros F.C., Lambert A., Carvalho M. (2023). Machine learning for accurate estimation of fetal gestational age based on ultrasound images. NPJ Digit. Med..

[20] Wang Y., Shi Y., Huang H. (2023). Fetal weight estimation based on deep neural network: A retrospective observational study. BMC Pregnancy Childbirth.

[21] Kuhle S., Maguire B., Zhang H., Hamilton D., Allen A.C., Joseph K., Allen V.M. (2018). Comparison of logistic regression with machine learning methods for the prediction of fetal growth abnormalities: A retrospective cohort study. BMC Pregnancy Childbirth.

[22] Akhtar F., Li J., Pei Y., Imran A., Rajput A., Azeem M., Wang Q. (2019). Diagnosis and prediction of large-for-gestational-age fetus using the stacked generalization method. Appl. Sci..

[23] Iqbal F., Chandra P., Khan A.A., Lewis L.E.S., Acharya D., Vandana K., Jayashree P., Shenoy P.A. (2023). Prediction of mortality among neonates with sepsis in the neonatal intensive care unit: A machine learning approach. Clin. Epidemiol. Glob. Health.

[24] Qamar T., Bawany N.Z. (2023). Understanding the black-box: Towards interpretable and reliable deep learning models. PeerJ Comput. Sci..

[25] Chakraborty S., Tomsett R., Raghavendra R., Harborne D., Alzantot M., Cerutti F., Srivastava M., Preece A., Julier S., Rao R.M. Interpretability of deep learning models: A survey of results. Proceedings of the 2017 IEEE Smartworld, Ubiquitous Intelligence & Computing, Advanced & Trusted Computed, Scalable Computing & Communications, Cloud & Big Data Computing, Internet of People and Smart City Innovation (smartworld/SCALCOM/UIC/ATC/CBDcom/IOP/SCI).

[26] Liao W., Zou B., Zhao R., Chen Y., He Z., Zhou M. (2019). Clinical interpretable deep learning model for glaucoma diagnosis. IEEE J. Biomed. Health Inform..

[27] Ramchandani A., Fan C., Mostafavi A. (2020). Deepcovidnet: An interpretable deep learning model for predictive surveillance of covid-19 using heterogeneous features and their interactions. IEEE Access.

[28] Rudin C. (2019). Stop explaining black box machine learning models for high stakes decisions and use interpretable models instead. Nat. Mach. Intell..

[29] Roccetti M., Delnevo G., Casini L., Mirri S. (2021). An alternative approach to dimension reduction for pareto distributed data: A case study. J. Big Data.

[30] Zelaya C.V.G. Towards explaining the effects of data preprocessing on machine learning. Proceedings of the 2019 IEEE 35th International Conference on Data Engineering (ICDE).

[31] Chawla N.V., Bowyer K.W., Hall L.O., Kegelmeyer W.P. (2002). SMOTE: Synthetic minority over-sampling technique. J. Artif. Intell. Res..

[32] Lundberg S.M., Nair B., Vavilala M.S., Horibe M., Eisses M.J., Adams T., Liston D.E., Low D.K.W., Newman S.F., Kim J. (2018). Explainable machine-learning predictions for the prevention of hypoxaemia during surgery. Nat. Biomed. Eng..

[33] Sun Y., Shen Z., Zhan Y., Wang Y., Ma S., Zhang S., Liu J., Wu S., Feng Y., Chen Y. (2020). Effects of pre-pregnancy body mass index and gestational weight gain on maternal and infant complications. BMC Pregnancy Childbirth.

[34] Zhang S., Liu H., Li N., Dong W., Li W., Wang L., Zhang Y., Yang Y., Leng J. (2022). Relationship between gestational body mass index change and the risk of gestational diabetes mellitus: A community-based retrospective study of 41,845 pregnant women. BMC Pregnancy Childbirth.

[35] Li J., Pan Y., Zheng Q., Chen X., Jiang X., Liu R., Zhu Y., Huang L. (2024). Risk factors and glycaemic control in small-for-gestational-age infants born to mothers with gestational diabetes mellitus: A case–control study using propensity score matching based on a large population. BMJ Open.

[36] Hao Y., Qu L., Guo Y., Ma L., Guo M., Zhu Y., Jin Y., Gu Q., Zhang Y., Sun W. (2022). Association of pre-pregnancy low-carbohydrate diet with maternal oral glucose tolerance test levels in gestational diabetes. BMC Pregnancy Childbirth.

[37] Rong L., Hou N., Hu J., Gong Y., Yan S., Li C., Yang Z., Sun B. (2023). The role of TyG index in predicting the incidence of diabetes in Chinese elderly men: A 20-year retrospective study. Front. Endocrinol..

